# Supplementary material for: Tetraspanins from the liver fluke Opisthorchis viverrini stimulate cholangiocyte migration and inflammatory cytokine production
Source: Folia Parasitol. Author manuscript; Available in PMC 2025 Jul 1. (PMC12212972; doi:10.14411/fp.2023.017)
Supplement: Supplementary Material [file NIHMS2086107-supplement-Supplementary_Material.pdf]

Institute of Parasitology, Biology Centre CAS
Folia Parasitologica 2023, 70: 017: Supplementary file
Supplementary Fig S1.

Folia Parasitologica
http://folia.paru.cas.cz

Research Article OPEN ACCESS

Ruangsuwast A., Smout M.J., Brindley P.J., Loukas A., Laha T., Chaiyadet S. 2023: Tetraspanins from the liver fluke *Opisthorchis viverrini* stimulate cholangiocyte migration and inflammatory cytokine production. Folia Parasitol. 70: 017.

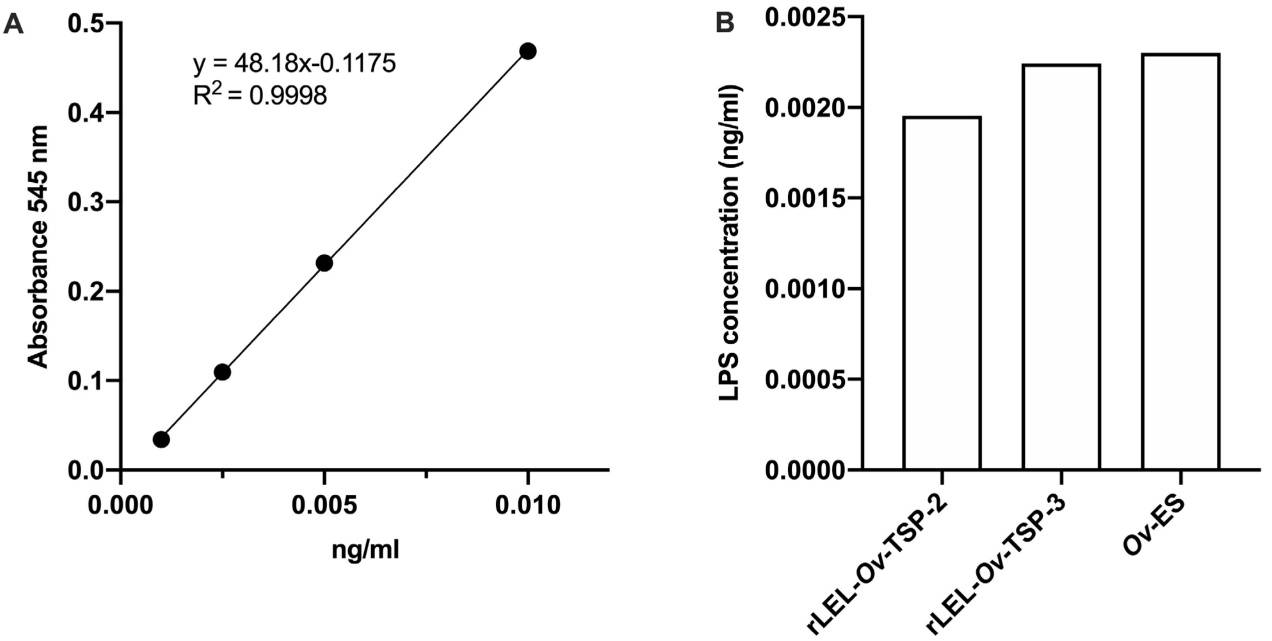

Fig. S1. A standard curve of endotoxin concentration at 0.01–0.001 ng/ml was developed using the Limulus amebocyte lysate (LAL) assay (A) to measure the endotoxin levels of rLEL-*Ov*-TSP2, rLEL-*Ov*-TSP-3, and *Ov*-ES (B).

Address for correspondence: Sujittra Chaiyadet, Department of Tropical Medicine, Faculty of Medicine, Khon Kaen University, Khon Kaen, 40002 Thailand. E-mail: sujitch@kku.ac.th

This is an Open Access article distributed under the terms of the Creative Commons Attribution License (http://creativecommons.org/licenses/by/4.0), which permits unrestricted use, distribution, and reproduction in any medium, provided the original work is properly cited.
